# Impact of Oil Spill Stress on Amino Acid Abundance in *Heterosigma akashiwo*

**DOI:** 10.3390/metabo16060361

**Published:** 2026-05-27

**Authors:** Dan Xue, Haohan Su, Jie Yu, Xiaowen Yang, Na Li, Shimeng Chen

**Affiliations:** 1 College of Marine Technology and Environment, Dalian Ocean University, Dalian 116023, China; 2 Department of Marine Engineering, Dalian Maritime University, Dalian 116026, China

**Keywords:** microalgae, amino acid, oil spill, biological metabolism

## Abstract

**Background**: Oil spills have dramatically increased, causing significant damage and pollution to marine ecosystems. The entry of petroleum hydrocarbons into the ocean may lead to the occurrence of harmful algal blooms (HABs). The amino acid changes in harmful algae after oil spills remain unclear. **Methods**: In order to study the effect of oil spills on the amino acid mechanism of typical causative species, the composition and relative abundance of amino acids in *Heterosigma akashiwo* were investigated under different water accommodated fractions (WAFs) of 180# fuel oil. **Results**: Random forest prediction of polycyclic aromatic hydrocarbon toxicity to microalgae identified pyrene, benzo[k]fluoranthene, and fluoranthene as significant contributors. A total of 16 species of amino acids were detected in *Heterosigma akashiwo*, among which alanine, proline, aspartic acid, cysteine, lysine, and histidine were the predominant ones. As the concentration of the WAF increased, alanine abundance decreased significantly, indicating that the WAF disrupted the metabolic balance of alanine, with the degree of interference being positively correlated with exposure concentration. With the increase in culture time, the abundance of cysteine increased at 1%, 3%, and 5% WAFs, whereas the cysteine increased and then decreased at 7% and 10% WAFs. The abundance of aspartic acid and lysine showed no obvious pattern with culture time under WAF stress. Significant increases in the abundance of proline and histidine were observed in the WAF treatments. **Conclusions**: This study investigated the impact of oil spill pressure on the amino acid content of harmful algae, providing a scientific basis for understanding the potential impact of oil spills on the occurrence of HABs.

## 1. Introduction

With the expansion of the population and the acceleration of economic growth, the pace of offshore oil exploration and development has surged dramatically. The continuous expansion of marine oil transportation and oil-related industries has led to a significant increase in the frequency of oil spills. A total of 1887 oil spills were documented as a result of ship collisions between the 1970s and 2020s. Among them, 480 were categorized as major oil spills, each involving the discharge of more than 700 t of crude oil [1]. The frequency of oil spills has caused great damage to marine ecosystems [2]. At present, there is no direct evidence to suggest a definite connection between PAHs and the formation of harmful algal blooms (HABs) [3]. However, HABs have been shown to occur in multiple areas after oil spills. For instance, previous studies reviewed 21 major oil spills worldwide, revealing that 11 of them led to HABs between 1967 and 2018 [4]. Among them, large blooms of heterotrophic dinoflagellates triggered HABs during the Deepwater Horizon oil spill in the Gulf of Mexico (2010) [5]. In recent years, *Heterosigma akashiwo* (*H. akashiwo*) has garnered significant attention due to its strong adaptability and ecological harmfulness in HABs in the South China Sea and the Yellow Sea of China. *H. akashiwo* is a harmful algae bloom species in the class of *Ochrophyta*. The water samples collected from the bloom area in Geoje, Korea, revealed that *H. akashiwo* was the most abundant operational taxonomic unit (OTU), accounting for 38.31% [6]. In addition, *H. akashiwo* can cause the death of fish, and the exact fish kill mechanisms are still being verified. Currently, the mechanisms of fish death caused by *H. akashiwo* have been summarized by scientists as mucus secretion, reactive oxygen species (ROS) production, toxin production, and hemolytic activity [7].

The common pollutants found in petroleum include asphaltenes, resins, aliphatic hydrocarbons, benzene, toluene, ethylbenzene, xylene, and polycyclic aromatic hydrocarbons (PAHs) [8]. The lipophilicity of PAHs enables them to penetrate biological membranes and tend to accumulate in organelles. This interferes with the normal functions and stability of cells [9,10]. Generally, the water accommodated fraction (WAF) in oil spills poses the most immediate toxic hazards to aquatic organisms [11]. The WAF is mainly composed of PAHs and styrene. These toxic substances can change the plankton community structure by inhibiting or promoting the growth of marine plankton [12].

Most of the existing studies focus on the relationship between the growth dynamics of *H. akashiwo* and environmental factors (such as nutrients, temperature, and salinity). However, the metabolic response of microalgae to oil spill stress remains poorly understood. Amino acids (AAs) are the key components of protein synthesis [13]. They are involved in vital biological processes, such as metabolism and energy production, DNA replication and repair, signal transduction, osmoregulation, and defense mechanisms [14]. Therefore, the composition and content of AAs can sensitively reflect the adaptive strategies of *H. akashiwo* in response to adverse conditions.

In the present study, the effects of different concentrations of the WAF on AA composition in *H. akashiwo* were investigated. The objectives of this study were to (1) analyze the composition and content of AAs in microalgae via the time-course dimension of oil spill stress; (2) explore the response of the relative abundance of the key AAs in microalgae under different concentrations of WAF stress. To the best of our knowledge, this study provides a new perspective on the ecotoxicological mechanisms of HABs induced by oil spills.

## 2. Materials and Methods

### 2.1. Microalgae Culture

The seawater culture of *H. akashiwo* was provided by the Institute of the National Marine Environmental Monitoring Center (Dalian, China). The seawater was filtered using a 0.2 μm Whatman glass-fiber filter (Whatman, Maidstone, Kent, UK) and subsequently sterilized at high temperature. The salinity and pH of seawater were 35‰ and 8, respectively. The microalgae were cultured in seawater with modified Conway medium (Table 1). The conical flask was kept in a constant temperature light incubator (MGC-450BP, Shanghai bluepard instruments Co., Ltd., Shanghai, China) with an illumination intensity of 60 µmol m^−2^ s^−1^ and a 12 h light and 12 h dark cycle. The temperature was set at 20 ± 1 °C. The algae were shaken three times a day to prevent the microalgae from sinking or adhering to the container walls. Subsequently, the microalgae with normal color, no sedimentation, and no adhesion to the container walls were selected for the subsequent experiments.

### 2.2. Preparation of Fuel Oil

Fuel oil #180 was obtained from China Marine Bunker (Petro China) Co., Ltd. (Dalian, China). The chemical characterization of 180# fuel oil was as follows: density (at 20 °C) of 985 Kg m^−3^; flash point of 73 °C; kinematic viscosity (at 50 °C) of 175 mm^2^ s^−1^; and sulfur content (by mass fraction) of 2%. The WAF of fuel oil was prepared by mixing fuel oil and sterilized seawater in a ratio of 1:9. The sterilized seawater and fuel oil were stirred using a magnetic stirrer for 24 h at room temperature. The speed of the magnetic stirrer was 900 rpm, which enabled it to create vortices with a depth ranging from 20% to 25%. The mixture was immediately transferred to a separating funnel and left to stand for 4 h until the separation of the aqueous phase and non-dissolved phase was achieved. The aqueous phase is here referred to as the stock solution of the WAF in saturation. Then, the aqueous phase was collected into amber glass bottles and stored at 4 °C in a dark place. The WAF concentration of the total petroleum hydrocarbon (TPH) was determined by ultraviolet spectrophotometry [16].

### 2.3. The WAF of Fuel Oil Treatments

The cell density of *H. akashiwo* inoculated in the experiment medium was approximately 10^5^ cells mL^−1^. The TPH concentration of the WAF used in the experiment was 4.97 mg L^−1^. The experiment set up a control group and 5 WAF treatment groups. The short-term exposure experiment on the WAF was conducted in a 1 L conical flask, each filled with 1 L of culture. A sterile WAF solution was added to achieve final WAF concentrations of 1%, 3%, 5%, 7%, and 10%, corresponding to 0.05 mg L^−1^, 0.15 mg L^−1^, 0.25 mg L^−1^, 0.35 mg L^−1^, and 0.5 mg L^−1^ of WAF concentrations. An amount of 1 mL of nutrients and vitamins was, respectively, added to the conical flasks (Table 1). Three parallel groups were set for each experimental treatment condition. During the experiments, the algae liquid was filtered by the Whatman GF/F fiber filtration membrane at 24 h, 48 h, 72 h, and 96 h. The filter membrane was placed in an oven at 60 °C and dried for 48 h. An amount of 50 mL of the filtered water sample was stored in a sealed colorimetric tube, and then the PAHs were extracted. A scalpel was used to separate the algae from the filter membrane, and the microalgae were subsequently collected.

### 2.4. Extraction and Derivatization of Amino Acids

L-AA standards (20 mg portion, Sigma Aldrich, Merck KGaA, Darmstadt, Germany,) or algae sample (40 mg portion) were placed in Pyrex test tubes. An amount of 2 mL of 6 mol L^−1^ hydrochloric acid was added to the test tubes and then purged with nitrogen (N_2_) for 1 min. After the air had been completely replaced by N_2_, the test tubes were sealed. The samples were hydrolyzed at 110 °C for 24 h. The acid extracts were harvested by centrifugation at 2000 rpm for 20 min. The supernatants were slowly poured into the cation exchange column and washed with 2 mL of a methanol/water mixture (8:1). Free AAs were extracted by 2 mL 4 mol L^−1^ NH_4_OH and dried by N_2_ flow at 80 °C. Tryptophan was not quantified in this study, as it is completely destroyed during acid hydrolysis conditions [17].

The AA derivatives were derived by the N-pivaloyl-isopropyl (NPP) ester derivatization process, mainly referring to Metges et al. [18]. The internal standard of this experiment was nor-leucine, which was added to free AAs. The free AAs were dissolved in 1 mL of esterification reagent and then heated at 100 °C for 1 h. Reaction products dried by N_2_ flow at 60 °C for 30 min. After cooling, each sample was added to 2 mL of dichloromethane. The mixture was deposited into the silica gel column dropwise and dried by N_2_ flow at room temperature. Subsequently, AA derivatives were dissolved in 200 μL ethyl acetate and stored at −20 °C. During the experiment, no protective measures were taken for cysteine (Cys), which would be lost due to hydrolysis. Therefore, the abundance of Cys in this study was only an estimated minimum value.

### 2.5. Analysis of Amino Acids

The AA derivatives were analyzed and identified by Gas Chromatography–Mass Spectrometry (GC-MS, Thermo Fisher Scientific, Waltham, MA, USA). A DB-5MS capillary column (60 m × 0.25 mm × 0.25 μm) was selected for the GC Column. He (99.999% purity) was used as the mobile phase carrier gas with a flow rate of 1–2 mL min^−1^. The programmed temperature for the GC was as follows: an initial temperature of 70 °C was held for 1 min, then increased to 220 °C at a rate of 3 °C min^−1^, followed by an increase to 300 °C at a rate of 10 °C min^−1^, and finally held at 300 °C for 8 min. The inlet temperature was set at 280 °C, followed by 1 μL of AA derivatives injected in a non-shunt mode. The MS conditions were EI ionization mode, and the electron energy was 70 eV. The ion source and transmission line temperatures were 230 °C and 250 °C, respectively.

Compared with the MS database of the National Institute of Standards and Technology (NIST), 16 kinds of AAs were identified. The AAs isolated by NPP derivatization were alanine (Ala), glycine (Gly), valine (Val), leucine (Leu), isoleucine (Ile), proline (Pro), aspartic acid (Asp), threonine (Thre), serine (Ser), methionine (Met), glutamic acid (Glu), phenylalanine (Phe), cysteine (Cys), lysine (Lys), histidine (His), and tyrosine (Tyr). The relative abundance of each AA was obtained by using the total ion flow diagram and the peak area explosion normalization method.

### 2.6. Detection of Polycyclic Aromatic Hydrocarbons in Seawater

The extraction and analysis methods for PAHs mainly followed the approaches described by Li et al. [19]. PAHs of the WAF were identified by matching retention times to 16 PAH standards. A total of 11 PAHs were detected in the water samples, namely naphthalene, 2-methylnaphthalene, 1-methylnaphthalene, 1,2-dihydroacenaphthylene, fluorene, phenanthrene, anthracene, fluoranthene, pyrene, chrysene, and benzo[k]fluoranthene. The relative abundance of each PAH was obtained by using the total ion flow diagram and the peak area explosion normalization method.

### 2.7. Statistical Analyses

Data was reported as means ± standard deviation (SD). One-way analysis of variance was used to separately assess the effects of culture time and WAF concentrations on the relative content of microalgae AAs. The significance level of the experimental results was set at *p* < 0.05. Where ANOVA revealed significant effects, pairwise comparisons were performed using Tukey’s HSD test. The regression equation between culture time and AA content was analyzed using linear correlation analysis, and Pearson’s correlation coefficient was obtained through the two-tailed test in bivariate correlation. The AAs analysis process was all conducted using SPSS Statistics 26 (IBM Corporation, Somers, NY, USA). To identify which PAHs were primarily responsible for the WAF, random forest classification was performed using the relative abundances of 11 PAHs as predictor variables and the WAF concentration (Ctrl, 1%, 3%, 5%, 7%, and 10%) as the response variable. The sample normalization was carried out using normalization by median. The mean decrease accuracy was calculated to quantify the importance of each PAH in the WAF concentrations. The random forest analysis process was conducted using MetaboAnalyst 6.0 (McGill University, Canada).

## 3. Results

### 3.1. The Petroleum Hydrocarbon Components in Water Samples

**Figure 1:**
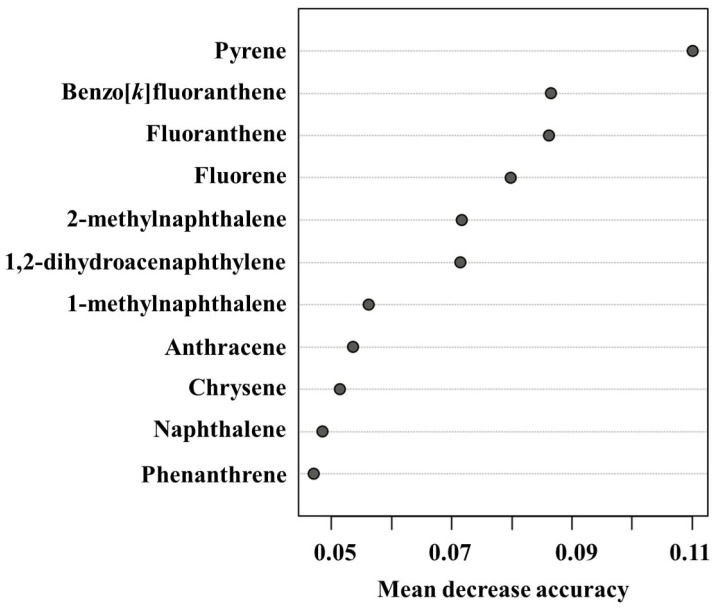
Dot plot showing the mean decrease accuracy for assessing the importance of each PAH feature.

Using the mean decrease in accuracy, pyrene was identified as the most important PAH, followed by benzo[k]fluoranthene and fluoranthene, with phenanthrene ranking eleventh (Figure 1). The significance of the 4-ring PAHs (pyrene and fluoranthene) was significantly higher than that of the 2–3 ring PAHs (naphthalene, phenanthrene, and anthracene). Therefore, these findings indicate that the PAHs in the 4–5 rings contribute more to the prediction of toxic effects on microalgae.

### 3.2. AA Composition of Heterosigma akashiwo

**Figure 2:**
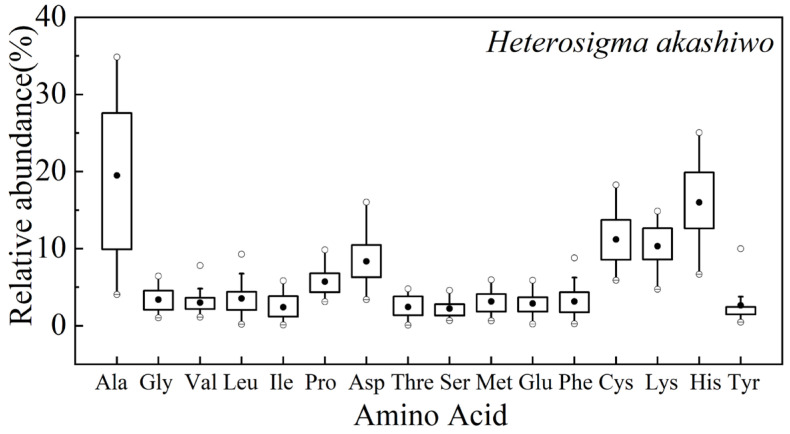
Composition of amino acids in the *H. akashiwo* acute toxicity experiment. The upper and lower whiskers indicate maximum and minimum values, the boxes show the 25th and 75th percentile range, and the line inside the boxes shows the median. The empty dots show outlier values, and the solid dots indicate average values.

As shown in Figure 2, 16 AAs were detected in *H. akashiwo*; namely, Ala, Gly, Val, Leu, Ile, Pro, Asp, Thre, Ser, Met, Glu, Phe, Cys, Lys, His, and Tyr. The relative abundance of six AAs was dominant, namely Ala, Pro, Asp, Cys, Lys, and His. Among them, Ala, Pro, Asp, and Cys are non-essential AAs, while Lys and His are essential AAs.

### 3.3. Toxic Effects of WAF on Ala and Cys in Microalgae

**Figure 3:**
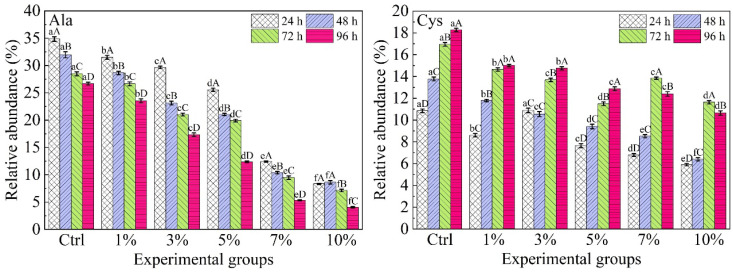
Effects of different WAF concentrations and culture time on the relative abundance of Ala and Cys in H. akashiwo. Error bars represent standard deviation (*n* = 3). The different lowercase letters (under the same culture time) and uppercase letters (under the same WAF concentration) on the column indicate significant differences (*p* < 0.05).

The effects of five different WAF concentrations on the relative abundance of Ala and Cys by microalgae are shown in Figure 3. In the control group, the relative abundance of Ala was decreased with increasing culture time, which decreased by 8.16 ± 0.17% at 96 h. On the contrary, the relative abundance of Cys increased with culture time. A significant correlation occurred between Cys abundance and the culture time (R = 0.987, *p* < 0.05, Table 2).

Under the different concentrations of WAF stress, the change trend of the relative abundance of Ala in *H. akashiwo* was the same as that in the control group. The Ala content decreased with prolonged culture time under different concentrations of the WAF. Except for the 10% WAF concentration, the Ala content showed a linear decrease within 96 h (R = −0.997, −0.976, −0.960, and −0.960 in the 1%, 3%, 5%, and 7% groups, *p* < 0.05, Table 2). Compared to the control group, the decrease in the abundance of Ala was 84.83% in microalgae exposed to 10% WAF concentration at 96 h.

The relative abundance of Cys increased with culture time at 1% and 5% WAF concentrations (Figure 3). The Cys content showed a time-dependent increase under 1% and 5% WAF concentrations (R = 0.957 and 0.997, *p* < 0.05, Table 2). In addition, it was observed that the Cys abundance increased over time at the 3% WAF condition, although this increase did not reach statistical significance (R = 0.919, *p* > 0.05, *n* = 4). The relative abundance of Cys increased and then decreased over the culture time in the 7% and 10% WAF concentrations. As the concentration of the WAF increased, the content of Cys showed a decreasing trend.

### 3.4. Toxic Effects of WAF on Asp, Lys, and Pro in Microalgae

**Figure 4:**
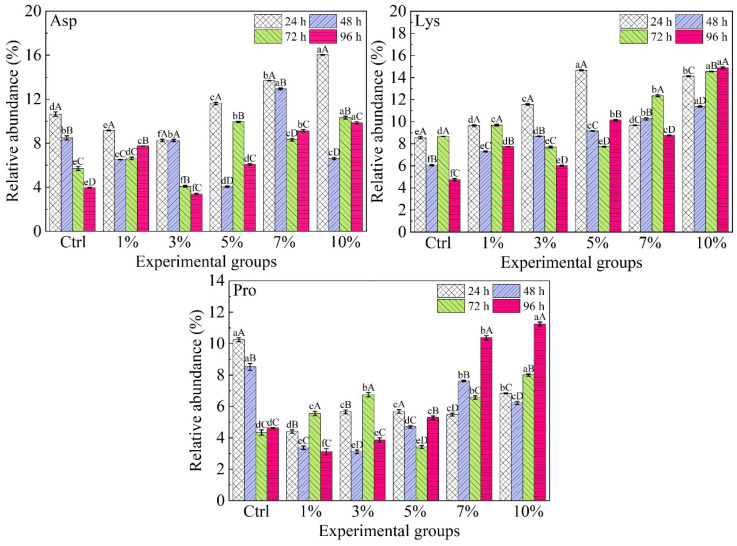
Effects of different WAF concentrations and culture times on the relative abundance of Asp, Lys, and Pro in *H. akashiwo*. The meaning of the letters can be found in Figure 3.

The relative abundance of Asp decreased with increasing culture time in the control group (R = −0.996, *p* < 0.01, Table 2). Under the different WAF conditions, the relative abundance of Asp at 24 h culture time was significantly higher than that at other culture times (*p* < 0.05). In addition, the relative abundance of Lys in *H. akashiwo* had no obvious regularity with culture time or WAF concentrations (Figure 4).

The relative abundance of Pro decreased during the period of 24 h to 72 h, and it stabilized around 72 h to 96 h in the control group (Figure 4). The relative abundance of Pro showed a nonlinear relationship with the culture time at 1%, 3%, and 5% WAF concentrations (Table 2). On the contrary, the relative abundance of Pro showed an upward trend in the 7% and 10% WAF concentrations (Figure 4).

### 3.5. Toxic Effects of WAF on His in Microalgae

**Figure 5:**
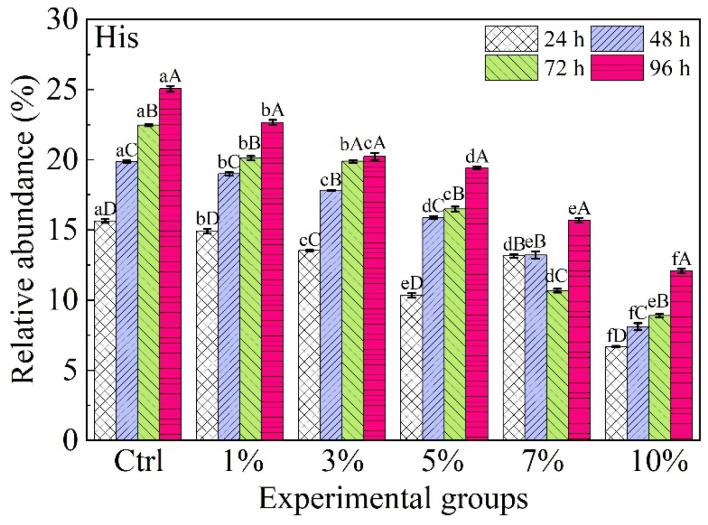
Effects of different WAF concentrations and culture times on the relative abundance of His in *H. akashiwo*. The meaning of the letters can be found in Figure 3.

Figure 5 shows the relative abundance of His in *H. akashiwo* when exposed to different concentrations of the WAF during a period of 24 h to 96 h. For the control group, the relative abundance of His exhibited increasing levels with increasing culture time. Also, His content was linearly correlated with the culture time (R = 0.991, *p* <  0.01). The relative abundance of His increased with culture times under 1%, 3%, 5%, and 10% WAF concentrations, whereas the abundance of His showed no regularity with exposure to the 7% WAF concentration. In addition, the relative abundance of His decreased significantly with the increase in WAF concentration except for 24 h (*p*  <  0.05).

## 4. Discussion

### 4.1. The Amino Acid Composition of Microalgae

Microalgae are an important source of marine AAs, providing AAs to the ocean through extracellular release and decomposition of debris [20]. Previous studies analyzed the AA composition of six species of dinoflagellates. The proportions of Glu, Asp, Arginine, Ala, Leu, and Thre were the highest [21]. In addition, the AA composition of 56 species of microalgae (belonging to seven phyla) was Ala, Gly, Ser, Pro, Asp, Glu, Cys, Tyr, Arg, Val, Leu, Thre, Ile, Met, Phe, Lys, His, and Tyr, respectively. The abundance values of Ala, Asp, and Glu were higher in non-essential AAs, while the abundance values of Leu, Phe, and Lys were higher in essential AAs [22]. The abundance of non-essential AAs in microalgae is dominant, which is an important source of AAs for heterotrophic organisms in the ocean [23]. Therefore, the composition and abundance of AAs in microalgae changed, which could directly affect the process of the marine ecological circulation system.

### 4.2. Toxic Effects of WAF on AAs in Microalgae

Previous studies have shown that the cell density of *H. akashiwo* increases as culture time extends. Moreover, the growth inhibition rate becomes greater with increasing WAF concentration [19]. Under high WAF stress, the changes in amino acid relative abundance may be influenced to some extent by cell viability and intracellular adaptive metabolism. Therefore, based on the results of this experiment and the AA metabolism processes described in published references, the influence of the WAF on the AA metabolism of microalgae is speculated through discussion.

Microalgae can synthesize the necessary AAs on their own to build proteins and other compounds. AAs are primarily synthesized through three pathways, namely reductive amination of α-keto acids, transamination, and interconversion of AAs. The precursors of Ala and Cys are pyruvate, which is produced by the glycolytic pathway. The carbon skeletons of Asp, Lys, and Pro are all derived from intermediates of the tricarboxylic acid (TCA) cycle, and are subsequently synthesized via dedicated amino acid biosynthetic pathways. His is obtained through the pentose phosphate pathway. This study classified and discussed the AAs that are part of the same metabolic pathways or reaction precursors.

#### 4.2.1. Alanine and Cysteine

Ala is synthesized mainly through the pyruvate pathway. Based on the substrate concentration and metabolic action, Ala and pyruvate are mutually converted through a reversible transamination reaction [24]. The precursor of acetyl-CoA is also pyruvate, which is involved in the TCA cycle. According to the experimental results of the Ctrl group, the relative abundance of Ala in microalgae decreases over time. During the exponential growth phase, the energy supply rate of the TCA cycle increases. Microalgae may extensively break down Ala to release the carbon skeleton, providing precursors for acetyl-CoA. On the contrary, the Cys relative abundance in *H. akashiwo* increased with culture time in the control group. Cys is the first stable organic sulfur compound formed by cells during the process of nutrient absorption. During this period, the sulfate absorbed during the cell growth phase was largely converted into Cys and stored [25].

Our results also found that the decrease in Ala and Cys relative abundance in *H. akashiwo* treated with WAF stress was noticed to be significant with respect to the control group (*p* < 0.05). The relative abundance of Ala and Cys overall decreased with the increase in WAF concentrations. The photosynthesis of microalgae is inhibited under the influence of PAHs, resulting in a reduction in carbon fixation products [26]. The decrease in glycolysis flux leads to a decrease in pyruvate content, which in turn affects the downstream synthesis of Ala and the supply of acetyl-CoA [21]. These findings indicate that the WAF may have interfered with the pyruvate metabolism of the microalgae. In addition, Ribulose-1,5-bisphosphate carboxylase/oxygenase (Rubisco), a key enzyme for CO_2_ fixation in microalgae [27], contains numerous Cys residues essential for its catalytic activity [28]. The observed upregulation of Cys at lower WAF levels may reflect increased demand for Rubisco synthesis as a compensatory response to photosynthetic stress. However, at higher WAF concentrations (7% and 10%), the subsequent decline in Cys abundance suggests that the toxic substances may eventually overwhelm this compensatory capacity, simultaneously impairing CO_2_ fixation efficiency and disrupting sulfur metabolism.

#### 4.2.2. Aspartic Acid, Lysine, and Proline

Asp demonstrates multifunctionality within biological systems. It is not only an essential component of protein synthesis but also plays a role in numerous metabolic pathways [29]. The experimental results revealed that the relative abundance of aspartic acid decreased as the culture time increased in the control group. This reduction is likely due to the fact that Asp, as an intermediate of the reaction, is involved in the synthesis of other AAs. Because of the complex reaction path of Asp, the Asp abundance showed no discernible pattern of change over the culture time under WAF stress. Previous studies found that the Asp metabolic pathway in *Euglena gracilis* did not show significant changes under antibiotic paromomycin stress, but it significantly changed under the influence of heavy metals (Cd) [30]. This further demonstrated that the changes in Asp content are controlled by multiple factors. Furthermore, the main pathway for Lys synthesis in microalgae cells primarily relies on the Asp pathway. In brief, Asp serves as a precursor that is converted into diaminopimelic acid (DAP), which is then transformed into Lys through a series of enzymatic reactions [31]. Lys has a complex reaction pathway and participates in other AA reactions. For this reason, there is no obvious regular relationship between the Lys content and the culture time or WAF concentrations.

Pro is a typical organic osmolyte in phytoplankton [32]. In this study, we found that the Pro abundance exhibited fluctuating patterns at lower WAF concentrations (1%, 3%, and 5%), while the Pro abundance in *H. akashiwo* increased with time at higher WAF concentrations (7% and 10%). By employing metabolomics, previous studies have identified that triphenyl phosphate exposure perturbs the metabolism of *Scenedesmus obliquus*. There were no significant differences in Pro observed in the control, 10 μg L^−1^, 100 μg L^−1^, and 1 mg L^−1^ triphenyl phosphate exposure groups in *Scenedesmus obliquus*. However, the significant increase in proline at the concentration of 10 mg L^−1^ triphenyl phosphate in *Scenedesmus obliquus* may reveal promoted osmoregulation [33]. Furthermore, as the WAF concentrations increased, the relative abundance of Pro increased at 96 h. Significant differences (*p*  <  0.05) in the abundance of Pro were observed between the treatment and control groups. The Pro level in the *Chlamydomonas reinhardtii* cells of the mothbean P5CS gene was 80% higher than that in the wild type algae, and it grew more rapidly at a Cd concentration of 100 μM. The higher glutathione levels in P5CS-expressing cells are believed to promote the formation of cadmium thiolate complexes in vacuoles, thereby playing a role in resisting heavy metal stress [34]. Recent studies have revealed that Pro produced under stressful conditions can act as an activator of reactive oxygen species (ROS) detoxification pathways [35,36]. Therefore, we supposed that microalgae significantly increase the content of Pro in order to resist the toxicity of the WAF.

#### 4.2.3. Histidine

His can be synthesized intracellularly through the pentose phosphate pathway (PPP). Usually, phosphoribosyl pyrophosphate (PRPP) is first converted to histidinol, which is then further transformed into His under the energy supply of ATP [37]. Energy metabolism is the key process to maintain the growth of microalgae, especially PPP. The upregulation of gene expression involved in PPP by the toxic effect of Cd (II) has been reported in a study on the energy mechanism of a fungal–microalgae co-culture system under Cd (II) stress for 20 h [38]. This pathway provides large amounts of NADPH and transformation conditions for His synthesis. Furthermore, His enhanced the defense mechanism of fungi and microalgae against Cd (II) [39]. As a result, the increase in relative His abundance may be related to its role in protecting cells from damage caused by WAF stress damage.

## 5. Conclusions

The present study examined the impact of various WAF concentrations on AAs in *H. akashiwo*. The determination of AA relative abundance in *H. akashiwo* revealed that Ala, Pro, Asp, Cys, Lys, and His were the dominant AAs. The analysis of key AAs revealed that the WAF of the oil spill significantly altered the abundance of some AAs. In concrete terms, the change trend of the Ala relative abundance after WAF exposure was the same as that in the control group. With the increase in culture time, the abundance of Cys increased at 1%, 3%, and 5% WAF concentrations, whereas the Cys increased and then decreased at 7% and 10% WAF concentrations. Furthermore, the WAF treatments increased the relative abundance of Pro and His, which might be a way to protect cells from oxidative stress damage. This study investigated the changes in key AAs within harmful microalgae under oil spill stress, providing a theoretical basis for understanding how oil spills may lead to the occurrence of HABs.

## Figures and Tables

**Table 1:** Nutrient formula of the Conway medium. Conway nutrients and vitamins were prepared in 1000 mL of ultra-pure water, respectively [15].

Conway Nutrient	Chemical Additive	Dosage (g)
A	FeCl_3_·6H_2_O	5.2
	MnCl_2_·4H_2_O	1.44
	H_3_BO_3_	134.4
	EDTA	180
	NaH_2_PO_4_·2H_2_O	80
	NaNO_3_	400
B	ZnCl_2_	2.1
	CoCl_2_·6H_2_O	2.0
	(NH_4_)_6_Mo_7_O_24_·4H_2_O	0.9
	CuSO_4_·5H_2_O	2.0
	Vitamin B_12_	0.4 mg
**Vitamins**	Vitamin H	0.5 mg
	Vitamin B_1_	100 mg

**Table 2:** Regression equation and determination coefficient of amino acid relative abundance and culture time in *H. akashiwo*.

WAF	Regression Equation	Pearson Correlation Coefficient	Regression Equation	Pearson Correlation Coefficient
Ala			Cys	
ctrl	y = −0.117x + 37.485	R = −0.993 **	y = 0.106x + 8.598	R = 0.987 *
1%	y = −0.108x + 34.042	R = −0.997 **	y = 0.0919x + 6.999	R = 0.957 *
3%	y = −0.163x + 32.552	R = −0.976 *	y = 0.0616x + 8.775	R = 0.919
5%	y = −0.169x + 29.870	R = −0.960 *	y = 0.0740x + 5.918	R = 0.997 **
7%	y = −0.0923x + 14.925	R = −0.960 *	y = 0.092x + 4.858	R = 0.870
10%	y = −0.0560x + 10.624	R = −0.886	y = 0.0812x + 3.774	R = 0.862
Asp			Pro	
ctrl	y = −0.0952x + 12.916	R = −0.996 **	y = −0.0878x + 12.199	R = −0.931
1%	y = −0.0171x + 8.539	R = −0.431	y = −0.0070x + 4.529	R = −0.196
3%	y = −0.0780x + 10.679	R = −0.924	y = −0.0072x + 5.277	R = −0.135
5%	y = −0.0447x + 10.602	R = −0.399	y = −0.0104x + 5.390	R = −0.324
7%	y = −0.0761x + 15.583	R = −0.879	y = 0.0569x + 4.099	R = 0.838
10%	y = −0.0616x + 14.402	R = −0.487	y = 0.0626x + 4.324	R = 0.867
Lys			His	
ctrl	y = −0.0367x + 9.2031	R = −0.588	y = 0.128x + 13.061	R = 0.991 **
1%	y = −0.0137x + 9.4093	R = −0.339	y = 0.101x + 13.096	R = 0.975 *
3%	y = −0.0736x + 12.905	R = −0.978 *	y = 0.0923x + 12.336	R = 0.929
5%	y = −0.0624x + 14.166	R = −0.647	y = 0.116x + 8.580	R = 0.948
7%	y = −0.00270x + 10.430	R = −0.056	y = 0.0212x + 11.910	R = 0.320
10%	y = 0.0229x + 12.3530	R = 0.441	y = 0.0707x + 4.687	R = 0.960 *

* and ** mean *p* < 0.05 and 0.01. *p* < 0.05 and 0.01 indicate statistically and highly significant linear correlations between the two variables.

## Data Availability

The original contributions presented in this study are included in the article. Further inquiries can be directed to the corresponding author.

## Abbreviations

The following abbreviations are used in this manuscript:

WAF	Water accommodated fraction
PAHs	Polycyclic aromatic hydrocarbons
HABs	Harmful algae blooms
OUT	Operational taxonomic unit
AAs	Amino acids
TPH	Total petroleum hydrocarbon
NPP	N-pivaloyl-isopropyl
GC-MS	Gas chromatography–mass spectrometry
NIST	National Institute of Standards and Technology
TCA	Tricarboxylic acid
Rubisco	Ribulose-1,5-bisphosphate carboxylase/oxygenase
DAP	Diaminopimelic acid
ROS	Reactive oxygen species
PPP	Pentose phosphate pathway
PRPP	Phosphoribosyl pyrophosphate
